# Correct the Coagulopathy and Scoop It Out: Complete Reversal of Anuric Renal Failure through the Operative Decompression of Extraperitoneal Hematoma-Induced Abdominal Compartment Syndrome

**DOI:** 10.1155/2012/946103

**Published:** 2012-12-17

**Authors:** Paul B. McBeth, Michael Dunham, Chad G. Ball, Andrew W. Kirkpatrick

**Affiliations:** ^1^Department of Surgery, Foothills Medical Centre, University of Calgary, Calgary, AB, Canada T2N 2T9; ^2^Critical Care Medicine, Foothills Medical Centre, University of Calgary, Calgary, AB, Canada T2N 2T9; ^3^Regional Trauma Program, Foothills Medical Centre, University of Calgary, Calgary, AB, Canada T2N 2T9

## Abstract

We report two cases of extraperitoneal compression of the intra-abdominal space resulting in abdominal compartment syndrome (ACS) with overt renal failure, which responded to operative decompression of the extra-peritoneal spaces. This discussion includes patient presentation, clinical course, diagnosis, interventions, and outcomes. Data was collected from the patient's electronic medical record and a radiology database. ACS appears to be a rare but completely reversible complication of both retroperitoneal hematoma (RH) and rectus sheath hematoma (RSH). In patients with large RH or RSH consideration of intra-abdominal pressure (IAP) monitoring combined with aggressive operative drainage after correction of the coagulopathy should be considered. These two cases illustrate how a relatively benign pathology can result in increased IAP, organ failure, and ultimately ACS. Intervention with decompressive laparotomy and evacuation of clot resulted in return to normal physiologic function.

## 1. Introduction

The influence of raised intra-abdominal pressure (IAP) known as intra-abdominal hypertension is recognized as having effects on nearly all aspects critically ill patient physiology [1, 2]. The most extreme manifestation of intra-abdominal hypertension (IAH) is new onset organ failure in the setting of an IAP greater than 20 mmHg, which defines the abdominal compartment syndrome (ACS) [3]. While IAH and ACS were classically described after damage control surgery from trauma and patients undergoing massive fluid resuscitation [4–6], these entities have also been associated with many different clinical conditions of the critically ill. 

With an aging population and increasing use of anticoagulant and thrombolytic therapies, spontaneous hematoma formation within the extra-peritoneal abdominal spaces (both retroperitoneal and rectus sheath) are increasingly being recognized. Anatomically these spaces are contiguous and massive bleeding within one often communicates with the other justifying there consideration as an extra-peritoneal hematoma (EPH). Although IAH/ACS are classically thought to arise from increases in the peritoneal volume from edematous viscera, distended lumen, iatrogenic packing materials, and resuscitative ascites [2], extra-peritoneal compression from pathology of the abdominal wall may also compress the abdominal cavity and induce IAH/ACS. The World Society of the Abdominal Compartment Syndrome (WSACS) consensus definitions thus recognizes both retroperitoneal hemorrhage and rectus sheath hematoma as rare but known abdominal causes of primary IAH/ACS [3]. Thus, overt ACS is a rare complication of extra-peritoneal compression of the intra-abdominal space in such cases as retroperitoneal hematoma (RH) or rectus sheath hematoma (RSH). Classically both RH and RSH are managed conservatively with operative intervention discouraged. We herein report two cases of extra-peritoneal compression on the intra-abdominal space resulting in an ACS with overt renal failure, which responded completely to operative decompression of the extra-peritoneal space. The University of Calgary Institutional Review Board approved this research.

## 2. Case Presentation

### 2.1. Case  1 

#### 2.1.1. Presenting History

**Figure 1:**
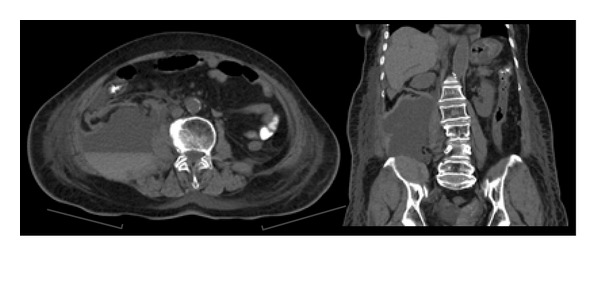
CT retroperitoneal hematoma (Case  1)—a large extraperitoneal hematoma measuring 18.3 × 11.2 cm arising from the posterior surface of lower part of left rectus abdominis muscle and extending into the pelvis causing right sided displacement of pelvic organs.

**Figure 2:**
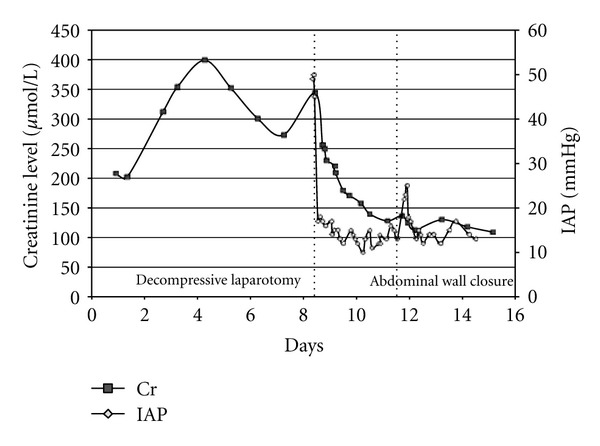
IAP and creatinine profiles (Case  1).

A 79-year-old female presented with nephrotic syndrome and underwent a right renal biopsy which confirmed Minimal Change Disease. Two days following the biopsy she developed hypovolemic shock with a hemoglobin level of 38 g/L and severe lactic acidosis. After resuscitation she underwent a CT scan revealing a large right RH (Figure 1). The patient subsequently developed ACS with IAP ranging from 40–50 mmHg and acute renal failure requiring continuous renal replacement therapy (Figure 2). Given the overt ACS she was taken to the operating room for an urgent decompression.

#### 2.1.2. Operative Findings and Intervention

Her operative therapy was staged as an initial decompressive laparotomy resulting in improved IAP, ventilation, and kidney function. Two days later the patient was taken back to the operating room for removal of the retroperitoneal clot and abdominal wall closure (Figure 3—video of clot removal available at: http://www.traumacanada.org/Default.aspx?pageId=829763). The retroperitoneal clot measured approximately 2.5 L. Postoperatively her kidney function returned to normal and ventilator support was no longer required.

### 2.2. Case  2

#### 2.2.1. Presenting History

A 71-year-old female presented initially with a right frontal lobe cerebrovascular accident with a suspected right common carotid artery clot. She was started on a heparin stroke protocol infusion and a phenylephrine infusion for systolic blood pressure goals of 170–180 mmHg. One week following her admission she started complaining of increasing abdominal pain. Examination revealed a mass in her right lower quadrant extending to her left lower quadrant. A subsequent CT scan and blood work demonstrated a RSH with hemoglobin drop to 52 g/L. Her anticoagulation was reversed with vitamin K and Fresh Frozen Plasma, however she continued to show evidence of ongoing bleeding. IAP monitoring revealed pressures averaging in the 40's and as high as 60 mmHg. She also developed acute renal failure secondary to an obstructive uropathy with evidence of hydronephrosis demonstrated on CT. The patient subsequently underwent an exploratory laparotomy and evacuation of her hematoma.

#### 2.2.2. Operative Findings and Intervention

**Figure 4:**
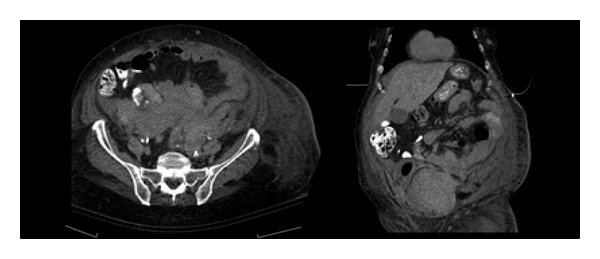
CT rectus sheath hematoma (Case  2)—a large retroperitoneal hematoma in the right posterior pararenal space, with an associated intramuscular hematoma in the right iliacus and right psoas muscle.

**Figure 5:**
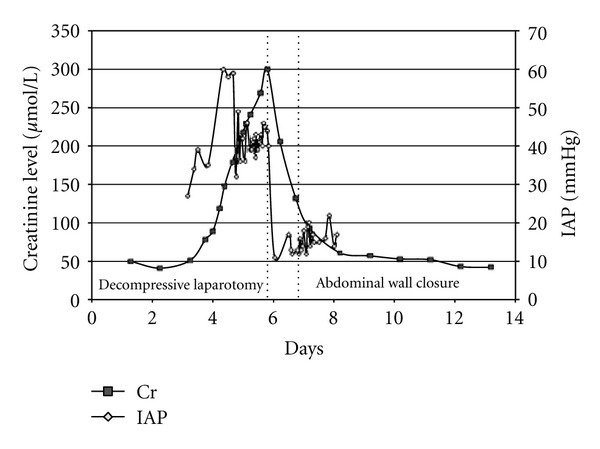
IAP and creatinine profiles (Case  2).

A decompressive laparotomy was undertaken revealing a massive extraperitoneal hematoma from the rectus sheath extending to the left hemipelvis (Figure 4). A 1.5 L hematoma was evacuated. The inferior epigastric vessel continued to bleed and was cauterized then over sewn. Attempts to place bilateral ureteral stents were made however the right-sided stent placement was unsuccessful due to technical difficulties. Post-operatively IAP improved in addition to ventilation and kidney function (Figure 5).

A second exploratory laparotomy was conducted 24 hrs later. The remainder of the hematoma cavity posterior to the left rectus muscle was irrigated and suctioned out. There was no evidence of any ongoing bleeding and the abdominal wall was closed. 

## 3. Discussion

With increased use of anticoagulant and thrombolytic therapies RH and RSH are becoming more prevalent. The management of both is traditionally conservative with correction of the coagulation profile and avoidance of invasive interventions [7]. Both RH and RSH are believed to be extremely rare causes of extra-peritoneal compression of the intra-abdominal space causing ACS. On rare occasions RH or RSH may expand enough to cause mass effect on the intra-abdominal space, or decreased abdominal wall compliance, leading to increased IAP and the potential for ACS. 

### 3.1. Retroperitoneal Hematoma

RH is a rare clinical finding typically associated with blunt or penetrating trauma to the aorta, kidney, pancreas, duodenum, or pelvis. Interventional procedures such as renal and bone marrow biopsies, ERCP and coronary angiography have been reported to cause retroperitoneal hemorrhage [8, 9] and resulting ACS [10]. Spontaneous RH is associated with Lenk's clinical triad (acute flank pain, symptoms of internal bleeding, and tenderness to palpation). Signs and symptoms of a RH include abdominal pain (67%), hematuria (40%), and shock (26.5%) [11–13]. Obstructive uropathy has also been described in patients with massive intrapelvic hematoma resulting in increased retroperitoneal pressure [14].

CT is considered the gold standard for the diagnosis of RH [12, 13, 15]. Therapeutic management after traumatic injury is guided by the anatomic division of the retroperitoneum into three zones (Table 1) and mechanism of injury. In penetrating trauma the majority of RH require exploration. The exception includes isolated lateral perirenal and pericolonic hematomas. Selective intervention is considered in patients with blunt trauma. Lateral perirenal and pelvic areas do not require operative management. Midline, lateral paraduodenal, lateral pericolonic, and portal hematomas are opened after proximal vascular control has been obtained [16]. The majority of spontaneous RH are managed conservatively however. Interventional measures are required with evidence of ongoing bleeding and hemodynamic instability. Embolization has developed as a promising less invasive method to control active hemorrhage [17]. Patients with ACS require a decompressive laparotomy in order to reverse their shock state.

### 3.2. Rectus Sheath Hematoma

RSH is also well recognized and described as case reports in the literature [19, 20]. This entity is caused by the rupture of rectus muscle fibers or a tear in the epigastric vessels which hemorrhage into the rectus sheath. Blunt trauma and anticoagulation are the most common causes however, spontaneous hematomas can occur due to coagulopathies, pregnancy, intra-abdominal injections, laparoscopic trocar placement, arteriovenous malformations, and even minimal trauma such as stretching or twisting of the abdomen [21–23]. A retrospective review from the Mayo Clinic demonstrated the majority (69%) of patients with RSH were undergoing anticoagulation therapy [24]. The incidence of RSH is low and accounts for 1.5–2% of cases of unexplained abdominal pain [24]. Clinical presentation may vary from minimal localized to severe diffuse abdominal pain. Physical examination may reveal a palpable, non-pulsatile, abdominal mass, and potential haemodynamic compromise. Other exam features include: Cullen's (periumbilical ecchymoses), Grey-Turner's (flank ecchymoses), Carnett's (abdominal pain remains unchanged or increases when the muscles of the abdominal wall are tensed), or Fothergill's (anterior abdominal mass does not cross the midline and is palpable when abdominal wall muscles are tensed) sign. RSH can be difficult to diagnose clinically and are frequently misdiagnosed [7, 25, 26]. CT and ultrasound are useful as diagnostic adjuncts and in the assessment of hematoma size and extent [27–29]. Berna has developed an RSH grading system (Grades I–III) based on CT imaging (Table 2) to guide therapeutic management [27]. The majority of RSH are treated conservatively with bed rest, analgesia, and correction of coagulopathy [30]. However, a small number of patients may develop IAH which may progress to ACS [31, 32]. Interventional measures may be required if there is evidence of continued bleeding. Percutaneous arterial embolization under radiological guidance has been successfully demonstrated [33, 34]. Surgical intervention is needed if the patient remains hemodynamically unstable or develops ACS. 

### 3.3. Abdominal Compartment Syndrome

IAP monitoring is important in critically ill patients because of the potentially fatal consequences of IAH and ACS. Physiological changes occur with increases in IAP that affect nearly every organ system. IAH and ACS are well described in the surgical literature and associated with primary intra-abdominal pathology and in patients undergoing large-volume resuscitation. The standard technique for IAP monitoring requires the use of an indwelling catheter manometry of the bladder.

Despite the rarity of recognized ACS from extra-peritoneal hematoma (EPH), limited precedents exist to support both operative intervention and evacuation of the hematoma after correction of the coagulopathy. The first known description of the ACS secondary to a rectus sheath hematoma was reported by O'Mara and colleagues in 2003 [30]. They noted complete resolution of the renal failure with operative evacuation of a large RSH. Similarly, Dabney and colleagues [18], also noted the immediate production of urine and overall physiological improvement. While simple decompression of the peritoneal cavity without hematoma evacuation may address cardiorespiratory issues, it may not be sufficient to correct the renal derangements. Conversely, Milanchi and colleagues noted the failure of simple evacuation, which required reoperation and formal evacuation within eight hours. Andrade and colleagues [35] also reported a case of RH with ACS in which they simply decompressed the peritoneum with a Bogotta bag, and subsequently closed the abdominal cavity over a month without reporting on the specific renal status. 

Patients who develop RH/RSH are typically elderly and/or suffering from multiple comorbid conditions that discourage aggressive therapies. Despite this, clinicians should recognize the potential of these extra-peritoneal conditions to restrict abdominal wall compliance and compress the peritoneal space thus reducing intra-abdominal volume and inducing severe IAH. In our experience both RH and RSH may induce overt ACS requiring staged decompressive laparotomies. Both patients developed ARF as a result of increased IAP. The abdominal wall release and removal of the hematoma in both cases resulted in reversal of the shock state and return of completely normal kidney function. 

Thus, we believe that any patient with RH or RSH warrants vigilant measurement of the IAP. Further, the recognition of IAH should mandate an aggressive management approach [30, 31, 36]. Early recognition is needed before the development of ACS leads to multiple system organ failure and death. Management of ACS varies with grade or severity. Conservative measures include the use of colonic and gastric decompression, sedation, and removal of intra-abdominal fluid. If conservative measures are unsuccessful treatment progresses to surgical decompression [32, 37, 38].

## 4. Conclusions

In conclusion, ACS appears to be a rare but completely reversible complication of both RH and RSH. Therefore, despite this infrequency, patients with large RH or RSH should have IAP monitoring, and aggressive operative drainage after correction of the coagulopathy if ACS is recognized. The cases presented herein illustrate how a relatively benign pathology can result in increased IAP, organ failure, and ultimately ACS. Management with a decompressive laparotomy and evacuation of clot results in return to normal physiologic function.

## Figures and Tables

**Figure 3:**
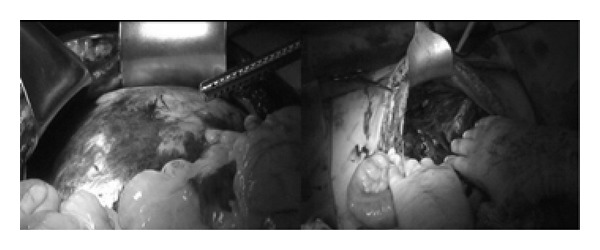
Intraoperative findings of a retroperitoneal hematoma causing increased intra-abdominal pressures.

**Table 1:** Diagnostic classification of retroperitoneal hematoma.

Zone	Description	Management
Zone I—midline retroperitoneum	Extends from the aortic hiatus to the sacral promontory and is divided into supramesocolic and inframesocolic zones	All injuries require surgical exploration

Zone II—lateral retroperitoneum	Extends on either side from the renal hila to the pericolic gutters Avoid exploration in blunt trauma	Explore all penetrating trauma

Zone III—pelvic retroperitoneum	Sacral promontory and encompasses the pelvis Explore all penetrating trauma	Explore for expanding hematoma in penetrating trauma

**Table 2:** Diagnostic classification of rectus sheath hematoma [18].

Grade	Description	Management
Grade I—mild	Intramuscular, unilateral, and does not dissect along fascia adjacent to the rectus muscle	Observation

Grade II—moderate	Intramuscular, dissects along adjacent fascia, may involve bilateral rectus muscles but without extension into the prevesical space	Anticoagulation reversal

Grade III—severe	Dissects along the fascia and extends into the peritoneum and the prevesical space.	Anticoagulation reversal Blood product administration

## References

[1] Ball CG, Kirkpatrick AW, McBeth P (2008). The secondary abdominal compartment syndrome: not just another post-traumatic complication. *Canadian Journal of Surgery*.

[2] Kirkpatrick AW, Balogh Z, Ball CG (2006). The secondary abdominal compartment syndrome: iatrogenic or unavoidable?. *Journal of the American College of Surgeons*.

[3] Malbrain ML, Cheatham ML, Kirkpatrick A (2006). Results from the International Conference of Experts on Intra-abdominal Hypertension and Abdominal Compartment Syndrome. I. Definitions. *Intensive Care Medicine*.

[4] Balogh Z, McKinley BA, Cocanour CS (2002). Secondary abdominal compartment syndrome is an elusive early complication of traumatic shock resuscitation. *American Journal of Surgery*.

[5] Fietsam R, Villalba M, Glover JL, Clark K (1989). Intra-abdominal compartment syndrome as a complication of ruptured abdominal aortic aneurysm repair. *American Surgeon*.

[6] Raeburn CD, Moore EE, Biffl WL (2001). The abdominal compartment syndrome is a morbid complication of postinjury damage control surgery. *American Journal of Surgery*.

[7] Berná JD, Zuazu I, Madrigal M, García-Medina V, Fernández C, Guirado F (2000). Conservative treatment of large rectus sheath hematoma in patients undergoing anticoagulant therapy. *Abdominal Imaging*.

[8] Neesse A, Kalinowski M, Walthers E, Görg C, Neubauer A (2009). Clinical management of massive retroperitoneal hemorrhage after bone marrow biopsy. *Leukemia and Lymphoma*.

[9] Tsai HL, Liu SW, How CK, Chern CH, Yen DHT, Huang CI (2008). A rare case of massive retroperitoneal hemorrhage after bone marrow aspiration alone. *American Journal of Emergency Medicine*.

[10] Milanchi S, Magner D, Lo SK, Klein AS, Colquhoun SD, Nissen NN (2007). Abdominal compartment syndrome secondary to retroperitoneal hematoma as a complication of ERCP after liver transplantation. *Transplantation Proceedings*.

[11] McDougal WS, Kursh ED, Persky L (1975). Spontaneous rupture of the kidney with perirenal hematoma. *Journal of Urology*.

[12] Morgentaler A, Belville JS, Tumeh SS, Richie JP, Loughlin KR (1990). Rational approach to evaluation and management of spontaneous perirenal hemorrhage. *Surgery Gynecology and Obstetrics*.

[13] Wolff JM, Jung PK, Adam G, Jakse G (1998). Spontaneous retroperitoneal haemorrhage associated with renal disease. *Journal of the Royal College of Surgeons of Edinburgh*.

[14] Hessmann M, Rommens P (1998). Bilateral ureteral obstruction and renal failure caused by massive retroperitoneal hematoma: is there a pelvic compartment syndrome analogous to abdominal compartment syndrome?. *Journal of Orthopaedic Trauma*.

[15] Kendall AR, Senay BA, Coll ME (1988). Spontaneous subcapsular renal hematoma: diagnosis and management. *Journal of Urology*.

[16] Feliciano DV (1990). Management of traumatic retroperitoneal hematoma. *Annals of Surgery*.

[17] Akpinar E, Peynircioglu B, Turkbey B, Cil BE, Balkanci F (2008). Endovascular management of life-threatening retroperitoneal bleeding. *ANZ Journal of Surgery*.

[18] Dabney A, Bastani B (2001). Enoxaparin-associated severe retroperitoneal bleeding and abdominal compartment syndrome: a report of two cases. *Intensive Care Medicine*.

[19] Dubinsky IL (1997). Hematoma of the rectus abdominis muscle: case report and review of the literature. *Journal of Emergency Medicine*.

[20] Siu WT, Tang CN, Law BKB, Chau CH, Li MKW (2003). Spontaneous rectus sheath hematoma. *Canadian Journal of Surgery*.

[21] Humphrey R, Carlan SJ, Greenbaum L (2001). Rectus sheath hematoma in pregnancy. *Journal of Clinical Ultrasound*.

[22] Ozaras R, Yilmaz MH, Tahan V, Uraz S, Yigitbasi R, Senturk H (2003). Spontaneous hematoma of the rectus abdominis muscle: a rare cause of acute abdominal pain in the elderly. *Acta Chirurgica Belgica*.

[23] Titone C, Lipsius M, Krakauer JS (1972). ‘Spontaneous’ hematoma of the rectus abdominis muscle: critical review of 50 cases with emphasis on early diagnosis and treatment. *Surgery*.

[24] Cherry WB, Mueller PS (2006). Rectus sheath hematoma: review of 126 cases at a single institution. *Medicine*.

[25] Edlow JA, Juang P, Margulies S, Burstein J (1999). Rectus sheath hematoma. *Annals of Emergency Medicine*.

[26] Maharaj D (2002). Rectus sheath haematoma: a new set of diagnostic features. *Postgraduate Medical Journal*.

[27] Berná JD, Garcia-Medina V, Guirao J, Garcia-Medina J (1996). Rectus sheath hematoma: diagnostic classification by CT. *Abdominal Imaging*.

[28] Huang MY, Chang WH (2009). Rectus sheath haematoma: over-diagnosis and under-diagnosis. *Emergency Medicine Journal*.

[29] Klingler PJ, Wetscher G, Glaser K, Tschmelitsch J, Schmid T, Hinder RA (1999). The use of ultrasound to differentiate rectus sheath hematoma from other acute abdominal disorders. *Surgical Endoscopy*.

[30] O’Mara MS, Semins H, Hathaway D, Caushaj PF (2003). Abdominal compartment syndrome as a consequence of rectus sheath hematoma. *American Surgeon*.

[31] Luhmann A, Williams EV (2006). Rectus sheath hematoma: a series of unfortunate events. *World Journal of Surgery*.

[32] Sieh KM, Chu KM, Wong J (2001). Intra-abdominal hypertension and abdominal compartment syndrome. *Langenbeck’s Archives of Surgery*.

[33] Rimola J, Perendreu J, Falcó J, Fortuño JR, Massuet A, Branera J (2007). Percutaneous arterial embolization in the management of rectus sheath hematoma. *American Journal of Roentgenology*.

[34] Zissin R, Gayer G, Kots E, Ellis M, Bartal G, Griton I (2007). Transcatheter arterial embolisation in anticoagulant-related haematoma—a current therapeutic option: a report of four patients and review of the literature. *International Journal of Clinical Practice*.

[35] Andrade MMDA, Pimenta MB, Belezia BDF, Xavier RL, Neiva AM (2007). Abdominal compartment syndrome due to warfarin-related retroperitoneal hematoma. *Clinics*.

[36] Orlando R, Eddy VA, Jacobs LM, Stadelmann WK (2004). The abdominal compartment syndrome. *Archives of Surgery*.

[37] Hunter JD, Damani Z (2004). Intra-abdominal hypertension and the abdominal compartment syndrome. *Anaesthesia*.

[38] Walker J, Criddle LM (2003). Pathophysiology and management of abdominal compartment syndrome. *American Journal of Critical Care*.

